# Use of medicine pricing and reimbursement policies for universal health coverage in Indonesia

**DOI:** 10.1371/journal.pone.0212328

**Published:** 2019-02-19

**Authors:** Riswandy Wasir, Sylvi Irawati, Amr Makady, Maarten Postma, Wim Goettsch, Erik Buskens, Talitha Feenstra

**Affiliations:** 1 Department of Epidemiology, University Medical Center Groningen, University of Groningen, Groningen, the Netherlands; 2 Sekolah Tinggi Ilmu Farmasi Makassar, Makassar, Indonesia; 3 Groningen Research Institute of Pharmacy, PharmacoTherapy, -Epidemiology & -Economics, University of Groningen, Groningen, the Netherlands; 4 Center for Medicines Information and Pharmaceutical Care, Faculty of Pharmacy, Universitas Surabaya, Surabaya, Indonesia; 5 Department of Clinical and Community Pharmacy, Faculty of Pharmacy, Universitas Surabaya, Surabaya, Indonesia; 6 National Health Care Institute, Diemen, the Netherlands; 7 Department of Pharmacoepidemiology and Clinical Pharmacology, Utrecht University, Utrecht, the Netherlands; 8 Department of Health Sciences, University of Groningen, Groningen, the Netherlands; 9 Department of Economics, Econometrics & Finance, Faculty of Economics & Business, University of Groningen, Groningen, the Netherlands; 10 Department of Pharmacology and Therapy, Faculty of Medicine, Universitas Airlangga, Surabaya, Indonesia; 11 Department of Operations, Faculty of Economics & Business, University of Groningen Groningen, the Netherlands; 12 Dutch National Institute for Public Health and the Environment, Bilthoven, the Netherlands; Jagiellonian University, POLAND

## Abstract

**Objectives:**

This study aimed to define the problems of the current use of the e-Catalogue and the national formulary (NF)—two elements of medicine pricing and reimbursement policies in Indonesia for achieving universal health coverage (UHC)—by examining the knowledge and attitudes of stakeholders. Specifically, to investigate (1) the perceived challenges involved in the further implementation of the e-Catalogue and the NF, (2) reasons of prescribing medicines not listed in the NF, and (3) possible improvements in the acceptance and use of the e-Catalogue and the NF.

**Methods:**

Semi-structured interviews were conducted with stakeholders (policymakers, healthcare providers, a pharmaceutical industry representative, and experienced patients) to collect the qualitative data. The data was analysed using directed content analysis, following the guidelines of the COnsolidated criteria for REporting Qualitative studies (COREQ) in reporting the findings.

**Results:**

Interestingly, 20 of 45 participants decided to withdraw from the interview due to their lack of knowledge of the e-Catalogue and the NF. All 25 stakeholders who fully participated in this research were in favor of the e-Catalogue and the NF. However, interviewees identified a range of challenges. A major challenge was the lack of harmonization between the lists of medicines in the e-Catalogue and the NF. Several system and personal reasons for prescribing medicines not listed in the NF were identified. Important reasons were a lack of incentives for physicians as well as a lack of transparent and evidence-based methods of selection for the medicines to be listed in the NF.

**Conclusions:**

The e-Catalogue and the NF have not been fully utilized for achieving UHC in Indonesia. Some possible improvements suggested were harmonization of medicines listed in the e-Catalogue and the NF, restructuring incentive programs for prescribing NF medicines, and increasing the transparency and evidence-based approach for selection of medicines listed in the e-Catalogue and the NF.

## Introduction

Many countries strive for universal health coverage (UHC), a concept recommended by the World Health Organization (WHO) to ensure that all people have access to health services they need without suffering financial hardship. One of the goals of UHC is to eliminate out-of-pocket payments (OOPs), a direct payment made by an individual to healthcare providers at the time of service use [1,2]. OOPs can be a great catastrophe for the family of a sick individual [3]. In low middle-income countries (LMICs), the proportion of OOPs in healthcare spending for medicines is high, ranging from 50% to 90% [4]. Moreover, medicine expenditure accounted for up to 67% of total health expenditure [5]. Appropriate policies are needed to reduce OOPs on medicines and to control medicine expenditure [6]. Furthermore, in order to move towards UHC, WHO has recommended to incorporate health technology assessment (HTA) to establish policies on medicines. The WHO defines HTA as the systematic evaluation of properties, effects and/or impacts of health technologies and interventions [7]. HTA is a critical component of evidence-based policy decision making [8,9].

In line with the global trends, Indonesia also makes a concerted effort to achieve UHC. Prior to 2014, Indonesians were supported by several social health insurances. The three biggest social health insurances were *Asuransi Kesehatan* (Askes, for civil servants and the military), *Jaminan Kesehatan Masyarakat* (Jamkesmas, for poor and near poor), and *Jaminan Sosial Tenaga Kerja* (Jamsostek, social security program for labourers). Notably, the fragmented health insurance schemes made health care spending and service quality difficult to control. Regarding medicines, previous studies have reported a large number of different medicine formularies existed with an unclear evidence-base [10]. In addition, OOPs were still dominant [11,12], e.g. the OOP in total was 49% in 2013 [13].

In 2014, the Government of Indonesia introduced a new health insurance scheme, known as *Jaminan Kesehatan Nasional–Kartu Indonesia Sehat* (JKN-KIS), which was managed by Indonesia’s National Healthcare Security Agency, namely *Badan Penyelenggara Jaminan Sosial*–*Kesehatan* (BPJS-Kesehatan). JKN-KIS was the result of a merger of pre-existing social health insurance schemes. Furthermore, the Ministry of Health (MOH) introduced several new medicine policies for supporting the implementation of the JKN-KIS program.

First, the e-Catalogue is a national medicine pricing policy and exists since 2013. The MOH proposes medicines, usually at the substance level (e.g. paracetamol), with specifications (dosage and types e.g. tablet, liquid, capsules) to LKPP (Lembaga Kebijakan Pengadaan Barang Jasa Pemerintah/ National Public Procurement Agency). Then, LKPP writes a tender for supply at a national scale and selects the preferred suppliers. As a result, the e-Catalogue provides a list of medicines with specifications, prices, and suppliers. All healthcare facilities are obliged to purchase medicines through the e-Catalogue. Nevertheless, healthcare facilities are allowed to perform their own tender for medicines that they need and are not listed in the e-Catalogue [14–16].

Second, the national formulary (NF) is a medicine reimbursement policy. It provides a list of medicines covered by BPJS-Kesehatan. The MOH has established the NF committee that is in charge of compiling the list of medicines in the NF. All healthcare facilities and healthcare professionals may propose medicines to be included in the NF. These medicines are then selected by the NF committee using several criteria among which efficacy, safety, marketing authorization, and benefit-risk ratio. In the NF, international non-proprietary names (INN) are used. The first NF was compiled in 2013 referring to formularies used in the previous health insurances in Indonesia and the last edition of the Indonesian essential medicines list. The list of medicines in the NF is revised at least every two years. The last revision was in 2017. All medicines listed in the NF should be available in the healthcare facilities. The director of the hospital and the hospital’s medical committee should approve when healthcare facilities prescribe medicines not listed in the NF. Notably, the approved medicine is not reimbursed by BPJS-Kesehatan [15,17,18], which implies either OOPs or payment from the hospital is needed.

Third, the use of HTA was introduced to assess new medicines which might have potential to be included in the e-Catalogue and the NF. The assessment is to be conducted by the HTA committee. This committee was formed by the MOH in April 2014, then renewed in 2016 [19,20]. The current HTA committee consists of eight senior health scientists and one employee of the MOH. They are supported by a technical staff (thirteen clinicians, two MOH employees, one engineer, and four secretaries). Currently, the main task of the HTA committee is to develop their program. The guidelines of the JKN-KIS state that the HTA committee is responsible for providing recommendations to the MOH regarding the list of healthcare services, including medicines, which are covered by the BPJS-Kesehatan. This implies the HTA committee is responsible for advice regarding the NF [15,20,21].

The Indonesian healthcare system since the implementation of the JKN-KIS shows several positive changes. Healthcare facilities seldom perform their own tender for procuring medicines [14]. Furthermore, instead of different formularies for each insurance scheme, there is a single NF. The MOH introduced the e-Catalogue and the NF, expecting that they would improve effectiveness, efficiency, and transparency in medicines procurement, as well as ensure the availability of good quality, efficacious, and affordable medicines [16,22]. Thus, this should help Indonesia to achieve UHC.

However, problems regarding the practical use of the e-Catalogue and the NF have been reported. For instance, it was found that many public healthcare facilites rarely use the e-Catalogue to procure medicines [23]. Moreover, of all medicines prescribed in 2015, up to 40% were not listed in the NF [24]. This may explain the increase of OOPs in 2015 to 50% [13]. Out of these, 70% were found to be OOPs for medicines [25], which obviously contrasts the philosophy of UHC. The obstacles hindering medicine pricing and reimbursement policies in Indonesia are far from clear and need to be investigated.

This research aims to shed light on the current use of the e-Catalogue and the NF by examining the knowledge and attitudes of stakeholders regarding the JKN-KIS program, especially concerning the e-Catalogue and the NF. Therefore, this research will investigate (1) the perceived challenges involved in the further implementation of the e-Catalogue and the NF, (2) the reasons behind the prescription of medicines not listed in the NF, and (3) possible improvements in the acceptance and use of the e-Catalogue and the NF.

## Methods

Semi-structured interviews were conducted to collect information about the perception of stakeholders regarding the JKN-KIS program, the e-Catalogue, and the NF. This study followed the reporting guidelines of the COnsolidated criteria for REporting Qualitative studies (COREQ) [26] to ensure good quality. The COREQ checklist provides guidance for explicit and comprehensive reporting of qualitative studies.

### Recruitment of participants

A purposive sampling method was used to recruit the stakeholders [27]. The following stakeholders in the healthcare system were approached to participate: policymakers (WHO members, NF committee, HTA committee, and JKN-KIS Agency), pharmaceutical industry, healthcare providers (physicians and pharmacists), and experienced patients. The stakeholders should have at least 5 years of relevant work experience. Experienced patients were selected based on the duration of their health insurance in Indonesia (at least 5 years) and their routine use of medicines (at least 5 years). In order to ensure that these patient criteria were met, participants were recruited from the BPJS-Kesehatan’s chronic disease management program (*Program pelayanan penyakit kronis*, Prolanis) [28]. The selection criteria of at least 5 years of experience for stakeholders was used to ensure that they could explain the healthcare system of Indonesia during and before the implementation of the new medicine policies.

A rough estimation of participants needed for attaining saturation was then made. A total of at least twenty-two stakeholders were aimed at. This was based on at least two representatives for each group of policy makers, at least two pharmaceutical industry representatives, at least four interviewees from each group of healthcare providers, and at least four patients. The reason for having more interviewees in the groups of healthcare providers and patients was our interest in the practical issues of the e-Catalogue and the NF, for instance prescribing medicines not listed in the NF.

### Interviews

**Fig 1:**
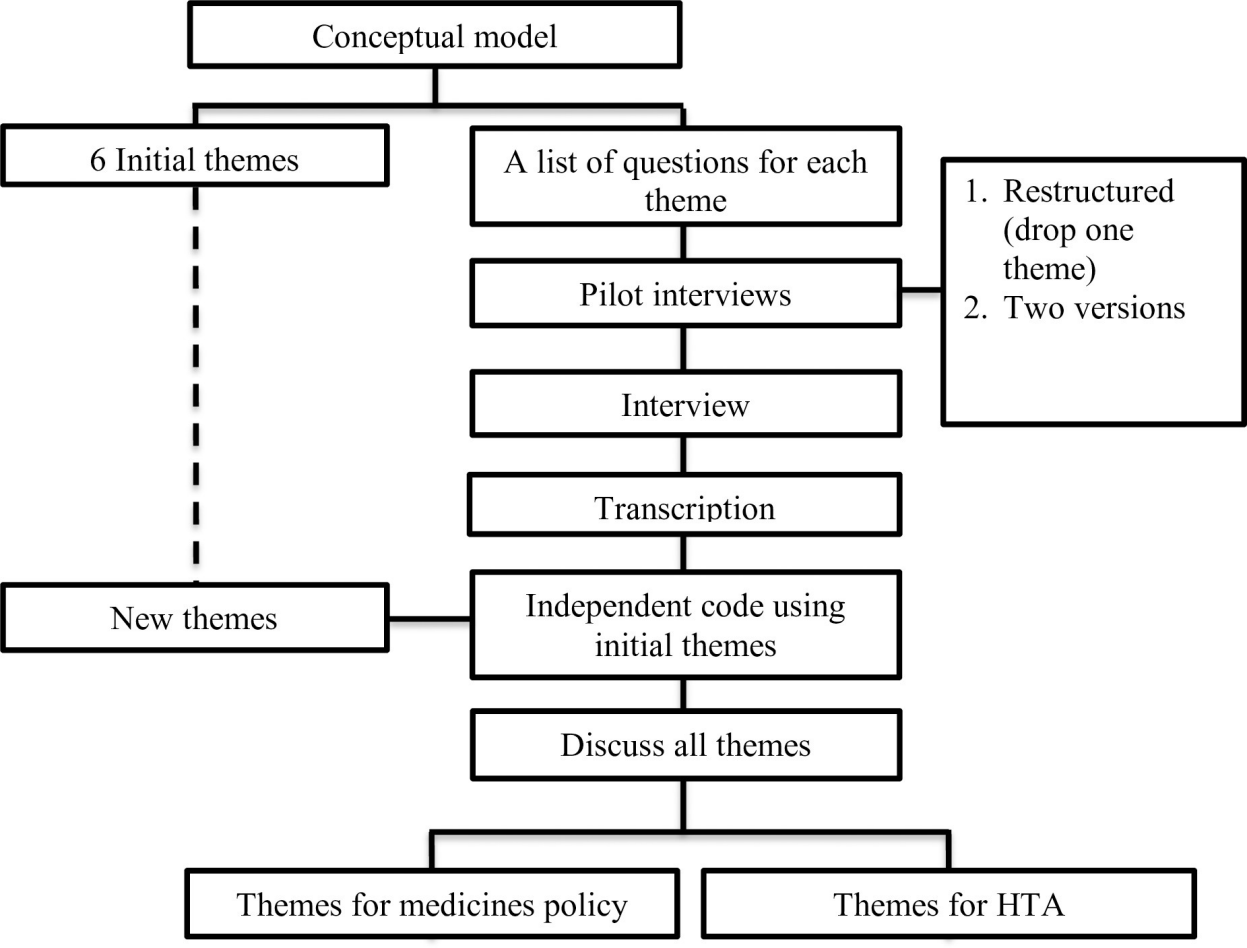
Research process.

An overview of the research process is provided in the flowchart (Fig 1). Five authors (RW, MP, WG, TF, and EB) first developed a conceptual model (S1 Fig), based on a review of WHO documents on UHC, and Indonesian documents regarding medicine policy and the use of HTA. As a result, the initial themes and a list of questions (S1 Table) for each theme were created, covering two research objectives, namely (1) the use of medicine pricing and reimbursement policy in the JKN-KIS program and (2) the barriers and facilitators of the application of HTA as part of this policy.

Pilot interviews were then conducted with nine stakeholders (pharmacists, physicians, and patients) with the aim of testing the comprehensibility and appropriateness of the initial themes and the list of questions. Some adjustments for the final list of questions were made, based on the pilot findings (S1 File), including skipping a theme and related questions into facts about health care costs that none of the pilot interviewees could answer. Most importantly, the list of questions was split into two versions. The first version was used for policymakers, the pharmaceutical industry, and healthcare providers, and the second version was used for patients. Patients in the pilot had less knowledge about the JKN-KIS program, the e-Catalogue, and the NF and therefore required a separate interview version.

The final list of questions was sent to the stakeholders in advance of the interview, in order to give interviewees some time for preparation. The interviews were recorded and subsequently transcribed verbatim by RW and SI for further analysis. The transcripts were sent back to the participants for feedback as a method to establish the credibility of the results (member checking). New interviews were conducted until no new issues arose, that is, until data saturation was obtained.

### Data analysis

Directed content analysis was conducted to systematically organize the qualitative data into a structured format [29] using MAXQDA version 12.3.2. The two Indonesian-speaking authors (RW and SI) read each transcript carefully. They then coded the transcripts independently using the initial themes. The codes were compared, and the non-fitting items were discussed. Texts that did not fit into one of the initial themes and were mentioned by several interviewees were coded as new themes. These new themes were then discussed with the research team (RW, SI, MP, AM, WG TF, and EB) to ensure consensus. RW and SI then checked saturation by theme. Saturation was reached when no new information was generated.

For the current study, four initial themes related to medicine policy were analyzed, namely, (1) the development of medicine policy and national health insurance, (2) medicine policy for UHC, (3) stakeholders’ perspective and (4) prescribing medicines not listed in the NF. The two remaining initial themes were left aside. The first of these, barriers and promoting factors of HTA, was analyzed in a separate study focusing on HTA. We skipped the final initial theme from the interviews, since eliciting the interviewees’ ideas regarding what conditions had the largest burden of disease and the amount of expenditure for medications, was impossible, as explained under interviews above.

### Ethical considerations

Written informed consent (S2 File) was obtained from all participants. Before the interviews, all participants were aware/understood that their participation was voluntary and that they were free to stop the interview at any time. All participants consented to review the verbatim transcript of their interview. The research plan and the interviews to be conducted were reviewed by the University Medical Canter Groningen (UMCG) ethical review board, who deemed the study non-intrusive, and subsequently provided a formal waiver statement, i.e., the study would not need regular ethical approval.

## Results

### Participants

Aiming at about 22 final participants, a total of 51 different individuals were approached during the recruitment process (S2 Table). Out of this number, 45 agreed to participate in this research. However, 9 withdrew after receiving list of questions; 7 decided to stop their interviews before they were finished, since they experienced difficulties in answering the questions and were not confident about their answers; and 4 had no time for an interview, leaving a final number of 25 participants. This means that the remaining interviewed persons were, to a certain degree, a select group with better knowledge than most stakeholders.

Classification of the twenty-five participants can be seen in Table 1. Participants included policy makers (WHO members, HTA Committee, NF Committee, JKN-KIS Agency), a medicine supplier (pharmaceutical industry), healthcare providers (physician and pharmacist), and users (patients). On average, the policymakers, the representative from the pharmaceutical industry and healthcare providers had a work experience of 26 years, with 14 years as the minimum. Furthermore, all patients interviewed had been enrolled in health insurance in Indonesia for an average of 11 years at the time of interview.

**Table 1:** Classification of participants involved in the study.

Participants	Gender	Age (years)	Work experience (years)	Years using public health insurance
**Policy makers**				
WHO Member	Male	50	25	-
HTA Committee	Female	53	28	-
HTA Committee	Male	55	30	-
NF Committee	Male	56	31	-
JKN-KIS Agency	Female	51	26	-
JKN-KIS Agency	Female	46	21	-
**Medicine supplier**				
Pharmaceutical Industry	Male	54	29	-
**Healthcare Providers**				
Physician	Male	58	33	-
Physician	Male	59	34	-
Physician	Male	66	41	-
Physician	Male	44	19	-
Physician	Male	51	26	-
Physician	Female	54	29	-
Pharmacist	Female	53	28	-
Pharmacist	Male	50	25	-
Pharmacist	Male	39	14	-
Pharmacist	Male	42	17	-
Pharmacist	Female	51	26	-
Pharmacist	Female	40	19	-
**Users**				
Patients	Male	53	-	8
Patients	Male	63	-	13
Patients	Female	59	-	10
Patients	Male	57	-	8
Patients	Female	63	-	12
Patients	Male	63	-	13

### Interviews and analysis of transcripts

Semi-structured interviews were conducted with stakeholders between August 2016 and April 2017 by RW. Interviews were conducted face to face (16 participants) or through video calls (9 participants) in locations comfortable to the participants. One of the interviewees invited her colleague to answer the questions together. One interview was conducted in English, while all others were in Bahasa. The average time spent on each interview was about an hour. All predetermined codes were covered by the interview results to a sufficient degree.

**Fig 2:**
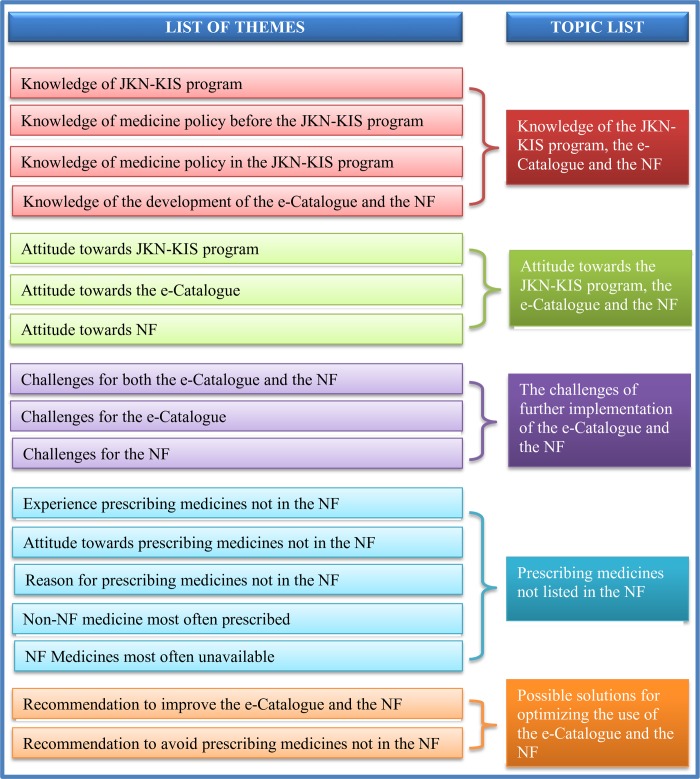
Clustering the themes.

Several texts from the transcripts could not be coded into one of the five initial themes (S1 Table), and were mentioned many times by different stakeholders. Therefore, 18 new themes identified by RW and SI were discussed with AM, WG, MP, TF, and EB to reach a consensus. Overall, 24 final themes were identified from the transcripts, of which 17 (S3 Table) were used in this study while the 7 remaining themes were left for further research and concerned HTA. The 17 themes in the current research were categorized into 5 topics, which were given overarching names (Fig 2).

#### Topic 1: Knowledge of the JKN-KIS program, the e-Catalogue and the NF

Among these 25 stakeholders, only patients had limited knowledge of the JKN-KIS program, the e-Catalogue, and the NF. The other stakeholders could explain the JKN-KIS program, the e-Catalogue, and the NF. Thus, to obtain accurate information from the patients for the next theme, the interviewer explained the JKN-KIS program to the patients, as well as the role and development of the e-Catalogue and the NF, based on the guidelines of medicines policies in Indonesia [14,16,22].

#### Topic 2: Attitude towards the JKN-KIS program, the e-Catalogue, and the NF

All stakeholders endorsed the aim of the JKN-KIS program to achieve UHC by 2020 and approved of the existence of the e-Catalogue and the NF. However, they emphasized that there were still some challenges to optimizing the implementation of these policies.

*“Yes*, *I agree*. *Why Universal Health Coverage by 2020*, *I hope it can be achieved next year (2017)*.*”* [Patient 3]

“*I agree with the existence of the e-Catalogue and the NF*. *However*, *the development and the implementation of these policies still need to be optimized*.” [Physician 3]

#### Topic 3: The challenges of further implementation of the e-Catalogue and the NF

One challenge for both the e-Catalogue and the NF that almost all stakeholders mentioned was the lack of harmonization between the list of medicines in the e-Catalogue and the NF. Some medicines in the NF are not listed in the e-Catalogue and vice versa. As a result, some NF medicines were in limited supply and difficult to provide and some were not even available at healthcare facilities. Furthermore, lack of harmonization between the e-Catalogue and the NF implied that healthcare facilities did not always use the e-Catalogue to procure medicines. Consequently, the prices of medicines were higher than needed.

“*If there were NF medicines that should be provided by the healthcare facilities*, *but these medicines were not in the e-Catalogue*, *this confused the medicine procurement committee*, *which would then have to spend time obtaining the standard price of the NF medicine*. *The price of medicines that has been listed in the e-Catalogue is much cheaper than previous price*” [Physician 2]

In fact, the Indonesian government allows the procurement of NF medicines not listed in the e-Catalogue with the approval of the hospital’s medical committee. However, approval often takes a long time.

“*To get claims from BPJS-Kesehatan*, *we ask the approval of the medical committee to help set the price*. *We usually wait for unpredictable lengths of time*. *It takes a long time to get approval*.” [Pharmacist 6]

A second challenge for both the e-Catalogue and the NF was the presence of a poor distribution system of medicines in combination with the geographical conditions of Indonesia. It is not easy to distribute NF medicines in a short period of time to all locations in Indonesia. This was mentioned in various ways by many participants.

*“Our country is vast*. *Distributing medicines in Jakarta will be different from distributing in Papua; the difficulties of distribution in Papua are great*. *Maybe the medicine is available*, *but it gets there too late*. *If the healthcare provider does not put the RKO (Plan of Medicine Needs) online*, *the pharmaceutical industry cannot anticipate demand*. *Now the pharmaceutical industry is trying to anticipate but it is too late*.” (Policymaker 4]

A challenge mentioned primarily by healthcare providers was that of the low price of medicines in the e-Catalogue. Consequently, some pharmaceutical industries were unable to provide NF medicines to healthcare facilities. If the pharmaceutical industry is pressed to provide the medicine at a low price, the quality of the medicine may also be lower. This is possible since no agency is responsible for controlling the good manufacturer practices (GMP) of pharmaceutical industry after they won the tender.

“*The price of the medicine set in the e-Catalogue is too low*. *So*, *they (the pharmaceutical industry) reduce their operating expenses*. *It is feared that economizing on operational expenses will put the quality of the medicines at risk*.” [Pharmacist 4]

*“The requirement to take part on a tender of medicines on the e-Catalogue is very simple*. *The most important thing is to have a GMP certificate*. *However*, *no one checked the pharmaceutical industry after they won tender*.*”* [Pharmaceutical Industry]

However, participants also stated that the low price of medicine in the e-Catalogue is caused by the pharmaceutical industry itself. Pharmaceutical firms bid low prices in order to win tenders and obtain the right to distribute medicines throughout Indonesia. Moreover, BPJS-Kesehatan was not involved in price-setting even though it is stated in the National Social Security System (SJSN) Law.

“*The price now deemed too low by the pharmaceutical industry is actually a result of competition among pharmaceutical firms regarding the supply of NF medicines through the e-Catalogue*. *So even though the estimated price was first set quite high–for example*, *paracetamol per tablet is set at a price of 70 rupiahs–because of the competition*, *an offer made was far below the price of 70*. *The goal of these firms is to win the tender*.” [Pharmaceutical Industry]

“*BPJS-Kesehatan is currently losing its power because of the tariff-setting by the Ministry of Health*. *In the National Social Security System (SJSN) Law*, *it is clear that the BPJS-Kesehatan is mandated to develop health system services and set prices and tariffs*.” [Policymaker 5]

Finally, it was observed that the NF was not the only reimbursement policy considered. For example, several hospitals have developed their own formularies. Such formularies list additional medicines not on the NF medicine list.

“*A hospital formulary is required by every hospital in Indonesia*. *The hospital formulary partially refers to the NF*. *For additional medicines not listed in the NF*, *patients do not have to pay*, *as long as the patients are participants in the JKN-KIS program and have the approval of the General Director of the Hospital*. *Those medicines will be subsidized by the hospitals*.” [Pharmacist 2]

The various challenges for further implementation of the e-Catalogue and the NF, mentioned in the interviews, are summarized in Table 2 and are categorized by policy types.

**Table 2:** Implementation challenges of the e-Catalogue and the NF.

Policies	Challenges
e-Catalogue	The price of medicines is set too low.
National Formulary	A lack of harmonization with other prescription guidelines (hospital formularies and standard treatment guidelines).
e-Catalogue and National Formulary	1. A lack of harmonization between the list of medicines in the e-Catalogue and the NF; 2. A poor distribution system for medicines and the geographical conditions of Indonesia.

#### Topic 4: Prescribing medicines not listed in the NF

All stakeholders were aware of the practice of prescribing medicines not listed in the NF. All physicians have prescribed medicines not listed in the NF, while all patients and other stakeholders have obtained prescriptions not listed in the NF.

“*Yes*. *Of course*. *There were still many prescriptions for medicines not in the NF; even I have prescribed medicines not listed in the NF*.*”* [Physician 5].

Almost all stakeholders were opposed to the prescription of medicines not listed in the NF. However, despite this reluctance, the stakeholders mentioned several reasons to explain this practice. Tables 3 and 4 list the main reasons mentioned as well as the underlying explanations.

**Table 3:** Reasons for prescriptions of medicines not listed in the NF in relation to the healthcare system.

Reasons	Causes
Unavailability of NF medicines in the healthcare facilities.	1. A lack of harmonization between the list of medicines in the e-Catalogue and the NF; 2. A poor distribution system; 3. The price of medicines in the e-Catalogue is too low;
Some important medicines are not listed in the NF.	1. Failure to negotiate the prices with the pharmaceutical industry; 2. Some importance medicines have not yet been registered in Indonesia;
The existence of other prescription guidelines.	The regulation of pharmaceutical service standards in hospitals requires a hospital formulary and standard treatment guidelines

**Table 4:** Reasons for prescriptions of medicines not listed in the NF in relation to individual stakeholders.

Reasons	Causes
Patients ask for non-NF medicines.	1. Patients do not know about the existence of NF medicines; 2. Patients perceive that NF medicines are low quality; 3. Patients are able to pay for non-NF medicines; 4. Patients prefer single combination medicine rather than taking separate medicines. 5. A lack of dissemination of NF to the healthcare facilities
Prescribers perceive no incentive to prescribe NF medicine.	Physicians have spent a significant amount of money and time on their training and expect to profit from working with the pharmaceutical industry.
Prescribers lack of confidence in the development of the NF.	1. The selection of medicines for inclusion in the e-Catalogue and the NF is not transparent and is not based on evidences; 2. Only a limited number of physicians are involved in the selection of medicines fo the e-Catalogue and the NF, thus does not represent all physicians.
Physicians do not know about the existence of the NF.	1. A lack of promotion of the NF, especially for new and resident physicians; 2. The habit of physicians to prescribe without references.
No sanction for physicians who prescribe non-NF medicines.	No system in place to check and fine.

The unavailability of NF medicines in healthcare facilities and its causes has been discussed above. Examples of several NF medicines that often prescribed but not available in healthcare facilities according to participating healthcare providers were ceftriaxone, cefotaxime, furosemide, metronidazole, lansoprazole, omeprazole, and paracetamol.

Medicines missing from the NF are another explanation for non-NF prescriptions. The list of non-NF medicines often prescribed in healthcare facilities according to healthcare providers and policymakerswere acetylcholine, amdixal, flexpen, flunarizine, glyceryl guaicolate, lincomycin, neurodex, novomix, pantoprazole, and piracetam.

“*Glyceryl guaiacolate is an important medicine for a coughing pregnant woman*. *This medicine is not in the NF*, *but its use is important*. *So*, *I still prescribe it if there are pregnant women who have a cough*.” [Physician 5]

Among these many reasons, the most interesting might be those relating to physicians’ perspective, since they write the prescriptions. Most of the physicians interviewed mentioned that their personal reason for prescribing medicines not listed in the NF was the lack of (financial) incentive.

“*Pity the doctor*, *once they were free to prescribe and get a bonus*. *Now they are limited by the NF*. *Many people insist on going to medical school*, *because hopefully*, *they will earn high salaries in the future*. *They will have paid expensive tuition fees*, *studied diligently*, *and lived less sociably because there was no time*. *But now their income is going to be limited*. *Pity the doctor*, *their individual rights were rescinded*, *for the benefit of society*.” [Physician 1]

Another reason mentioned by many physicians was lack of confidence in the development of the e-Catalogue and the NF.

“*I think the NF medicines were not selected on the basis of recent scientific evidences*. *I still suspect the selection of medicines was based on other considerations as well*. *For example*, *the pharmaceutical industry intervened so that certain medicines were included in the NF*. *In my opinion*, *at this time*, *the NF committee has not been able to account for the selection of medicines in the NF scientifically*, *because HTA is not yet running*.” [Physician 5]

“*The physicians disagreed with the list of NF medicines*. *Well*, *these complaints are usually from senior physicians who were not involved in drawing up the NF*.” [Physician 6]

In contrast to this, one of the policymakers stated that the list of medicines in the NF has, only to a certain degree, been selected based on evidences.

“*The medicine proposed by physicians for the NF must be supported by scientific evidence*, *so that the proposal is accountable*, *not just a proposal*.” [Policymaker 4]

#### Topic 5: Possible solutions for optimizing the use of the e-Catalogue and the NF

Some possible solutions were suggested by stakeholders to overcome the challenges of the e-Catalogue and the NF. Many participants (14 out of 25) recommended harmonizing the list of medicines in the e-Catalogue and the NF. Moreover, the selection of medicines needed to be transparent and evidence-based.

“*The government should harmonize all the medicines policies in the JKN-KIS system such as the NF*, *e-Catalogue*, *and also other medicine policies such as hospital formularies*, *standard treatment guidelines and Indonesian Case Base Groups (INA-CBGs)*” [Pharmacist 5]

*“The selection of medicines in the e-Catalogue and the NF should be transparent and reflect the latest scientific evidence*. *Therefore*, *HTA should be implemented*.” [Physician 5]

A second possible solution suggested was to apply rewards and punishments for prescribers in order to avoid prescriptions of medicines not listed in the NF. Almost all physicians and some policymakers mentioned this.

*“A bonus should be given to the physicians if they prescribe NF medicines*, *and consequently*, *the physicians will become more motivated*. *Furthermore*, *penalties can also be issued if the physicians prescribe medicines that are not in the NF*.*”* [Physician 1]

A third possible solution proposed was to have at least one distributor of NF medicines in all provinces in Indonesia, or alternatively, to have more than one industry providing each type of NF medicines. Some pharmacists and policymakers mentioned this.

“*There is a need for an alternative provider to the one who won the tender in the e-Catalogue*, *so that the price of more than one potential provider can be included for the same medicine*. *Thus*, *healthcare facilities would have alternatives when one of the pharmaceutical providers is unable to provide NF medicines*.” [Pharmacist 4].

A fourth possible solution recommended was to disseminate the list of NF medicines to patients. Furthermore, the list of NF medicines needed to be extended. Almost all patients and some policymakers mentioned this.

“*Every patient should receive a list of NF medicines*, *thus consequently being made aware of whether the medicine being administered to the patient is listed in the NF or not*. *The patient can then also ask for an NF medicine if his/her medicine is not included in the list of free medicines*.” [Patient 6]

“*The list of NF medicines should be expanded*, *so that all medicines would be free to the patients*.” [Patient 4]

A fifth possible solution was to ensure the quality of the list of medicines in the e-Catalogue and the NF. This possible solution was mentioned by the representative of the pharmaceutical industry.

“*Determining the winning bidder for NF medicines should not only be based on price and production capacity*. *There must be some kind of pre-qualification*. *There should be an audit to ascertain the quality of the medicines*, *which can be conducted using the WHO pre-qualification process*.” [Pharmaceutical Industry]

Another possible solution from policymakers was to encourage patients to report to the BPJS-Kesehatan when obtaining a prescription of medicines not listed in the NF.

“*Patients should give feedback on prescribed medicines not listed in the NF*. *In every health facility*, *there was a BPJS-Kesehatan office*. *So*, *if there is a complaint from the patient*, *we will confirm this with the doctor and pharmacist*.” [Policymaker 6]

## Discussion

All the stakeholders interviewed were in favour of the e-Catalogue and the NF. However, many healthcare providers and patients had limited knowledge of the role and the existence of the e-Catalogue and the NF. Interviewees identified a range of challenges preventing optimal implementation. The challenges mentioned were related to the general structure of the healthcare system as well as to individual behaviour. The stakeholders had the following suggestions for improvement: (1) The list of medicines in the e-Catalogue and the NF should be harmonized to accelerate the procurement of NF medicines and to facilitate the availability of NF medicines in healthcare facilities; (2) The procedure for selecting medicines in the NF and setting their prices in the e-Catalogue should be made more transparent and evidence-based in order to convince stakeholders to use them; (3) Physicians could be rewarded for prescribing NF medicines or sanctioned for writing prescriptions for medicines not listed in the NF; (4) More dissemination of information concerning the NF could be targeted to all citizens.

The suggestion to harmonize the e-Catalogue and the NF is related to the current confusion caused by a lack of harmony. For medicines on the NF, but not listed in the e-Catalogue, healthcare facilities need to find a reference price. However, the price must be in accordance with the maximum claims of the BPJS-Kesehatan. If healthcare facilities procure medicines at higher prices, healthcare facilities can lose money. Furthermore, the existence of individual hospital formularies adds to the confusion. Medicines that are not listed in the NF can be listed in a hospital formulary. A hospital formulary is needed for hospital accreditation [30,31]. Hence, here we see policies are not in harmony. Similar problems were noticed in other LMIC countries, e.g. Namibia, with different advices between different national bodies causing problems [32].

Lack of harmony was indicated by many interviewees. Therefore, the authors have compared both lists systematically. We found that in 2013 [33], there were 519 substances of medicines with 923 specifications in the NF, and 374 of the specifications were not listed in the 2018 e-Catalogue. In 2015 [34], 562 substances of medicines with 983 specifications were in the NF, and 359 of specifications were not listed in the e-Catalogue. In 2017 [35], 586 substances of medicines with 1031 specifications were in the NF, and 380 were not listed in the e-Catalogue. The e-Catalogue listed 1076 specifications of medicines. [36]. This highlight that the problem of lack of harmony was stable over time.

The current study identifies reasons for prescribing medicines not listed in the NF. A full list of the reasons for these non-NF prescriptions can be found in the results section. A first important reason is that physicians are not convinced by the listings in the NF, since the selection process was perceived as neither transparent nor evidence-based. The requirements linked to the successful implementation of a NF include transparency and an evidence-based selection process of the medicines [37]. Physicians trust in formularies is a key area [38]. One way to accomplish this might be the use of HTA, as also suggested by some interviewees. HTA has been used by many organisations in Economic Co-operation and Development (OECD) countries to select the medicines to be included in their NFs [39,40]. Furthermore, HTA can also facilitate the setting of reasonable prices for medicines [41]. Thailand is one of the Southeast Asian countries which is now successfully using HTA for selecting medicines in their medicine pricing and reimbursement list [6,42]. An HTA committee in Indonesia does exist, however, it is still in its early stages of development. Other Southeast Asian countries, such as the Philippines, Malaysia and Vietnam are also still in the early stages of HTA development [43–46].

A second important reason for prescribing non-NF medicines is the lack of incentives for the healthcare providers, particularly, the physicians who prescribe the medicines. A previous study suggested that providing incentives to the stakeholders could reduce the number of medicines not listed in the formularies [47]. Maybe the Indonesian government could rethink their policy regarding incentives for all stakeholders. The current regulations of Indonesia vis-à-vis rewards and punishments for healthcare professionals do not touch on this aspect of the prescription of medicines listed in the NF. Moreover, such clauses reward and punish only those physicians working in primary healthcare, not physicians working in hospitals [48]. In some countries, pay-for-performance schemes for providers have been set up. Such schemes could financially reward physicians for achieving healthcare system targets [49,50]. The literature discusses various methods to influence physicians’ prescribing, including incentives. Other methods consist of educational, organizational, and regulatory approaches [51,52].

The fourth suggestion to disseminate the NF to all healthcare facilities is equally important and touches upon the educational approach. Mostly patients had this suggestion since they were not aware of the existence of the NF. A previous study also suggested the quality of care to patients may be compromised by the lacking availability of some essential policy documents in the healthcare facilities [53]. Medicines listed in the NF are free of charge to patients. If patients are better aware of the list of medicines, they may avoid to use non-NF medicines. Therefore, this also can help to reduce OOPs, in particular for medicines.

Though a variety of stakeholders were interviewed in this study, this does not automatically mean that all stakeholders were represented. This research contained interviews with a specific group of patients, namely, those from Prolanis. The main reason for this was that participants from this group were relatively easy to find and recruit. Furthermore, we knew that at the time of the interviews, they had been taking medication since before the implementation of the JKN-KNS program in 2014, and therefore were able to provide informed views. This research highlighted a variety of barriers, facilitators and solutions which were mentioned by patients and professionals until saturation was reached. However, although more participants (25) were interviewed than the number of participants aimed at (22), this study did not manage to achieve two interviewees for each subgroup of stakeholders. Notably, only one WHO member, member of NF committee, and pharmaceutical industry representative were interviewed. Therefore, all policy makers were combined into one category. Nonetheless, the interviewees attained were relatively experienced. In particular, the representative of pharmaceutical industry interviewed was a leader in several pharmaceutical associations in Indonesia.

Conducting semi-structured interviews allowed the researchers to obtain detailed information about personal feelings, perceptions and opinions from the participants without any influences from other group members. Moreover, all ambiguities could be clarified, and incomplete answers were completely followed up. Several steps were taken to ensure good research practice during the data compilation and analysis stages. The sampling process used to select participants and the interview guide were compared with recommendations published in the COREQ [26]. Transcripts were coded independently by two authors and all discrepancies were resolved by consensus.

Some suggestions for policy improvement mentioned by the interviewees could facilitate the improvement of the e-Catalogue and the NF, specifically avoiding writing prescriptions for medicines not listed in the NF and setting reasonable prices in the e-Catalogue. These results could prove relevant to other countries striving to achieve UHC, particularly for the countries which are experiencing similar problems regarding the implementation of a medicine pricing and reimbursement policy.

## Conclusions

In conclusion, the e-Catalogue and the NF as medicine pricing and reimbursement policies of Indonesia have not been fully utilized to support the JKN-KIS program’s aim of achieving UHC. The main challenge identified by the interviewees was the lack of harmonization between the lists of medicines in the e-Catalogue and the NF. Important reasons mentioned for prescribing medicines not listed in the NF included a lack of incentives for physicians as well as a lack of transparent and evidence-based selection methods for the medicines to be included in the NF. Possible improvements to the use of e-Catalogue and the NF were discussed. First, the list of medicines in the e-Catalogue and the NF could be harmonized. Second, the current incentive program for providers could be altered to provide more rewards for prescribing NF medicines while more education on the NF would be worthwhile. Finally, the procedure for selecting medicines in the NF and setting their prices in the e-Catalogue could be made more transparent and evidence-based in order to convince stakeholders to use them.

## Supporting information

S1 Fig Conceptual framework. (PDF)

S1 Table Initial list of themes and of questions. (PDF)

S2 Table Overview of recruitment process. (PDF)

S3 Table Final list of themes and aim of the themes. (PDF)

S1 File Final list of questions. (PDF)

S2 File Informed consent. (PDF)

## References

[1] World Health Organization. The world health report: health systems financing: the path to universal coverage: executive summary. 2010. 10.2471/BLT.10.078741 PMC2878164 20539847

[2] World Health Organization. World health statistics 2016: monitoring health for the SDGs sustainable development goals.: World Health Organization; 2016.

[3] Cameron A, Ewen M, Ross-Degnan D, Ball D, Laing R. Medicine prices, availability, and affordability in 36 developing and middle-income countries: a secondary analysis. The lancet 2009;373(9659):240–249. 10.1016/S0140-6736(08)61762-6 19042012

[4] Bigdeli M, Laing R, Tomson G. Medicines and universal health coverage: challenges and opportunities. 2015. 10.1186/s40545-015-0028-4 PMC4350289 25825675

[5] Rickwood S, Kleinrock M, Nunez-Gaviria M, Sakhrani S, Aitken M. The global use of medicines: outlook through 2017 IMS Institute for Healthcare Informatics 2013:5.

[6] Wirtz VJ, Hogerzeil HV, Gray AL, Bigdeli M, De Joncheere CP, Ewen MA, et al Essential medicines for universal health coverage. The Lancet 2017;389(10067):403–476. 10.1016/S0140-6736(16)31599-9 PMC7159295 27832874

[7] World Health Organization. Health Technology Assessment, HTA, WHO Definition (EB 134/30). Access on January 5, 2019;.

[8] Chalkidou K, Marten R, Cutler D, Culyer T, Smith R, Teerawattananon Y, et al Health technology assessment in universal health coverage. Lancet 2013 12 21;382(9910): e48–9. 10.1016/S0140-6736(13)62559-3 24360390

[9] Angelis A, Lange A, Kanavos P. Using health technology assessment to assess the value of new medicines: results of a systematic review and expert consultation across eight European countries. The European Journal of Health Economics 2018;19(1):123–152. 10.1007/s10198-017-0871-0 28303438 PMC5773640

[10] Holloway KA. Pharmaceuticals in Health Care Delivery. Mission Report 2011;30.

[11] Kwon Soonman, Kim Sujin, Jeon Boyoung, Jung Youn. Pharmaceutical Policy and Financing in Asia-Pacific Countries.: OECD Korea Policy Centre, Graduate School of Public Health, Seoul National University (WHO Collaborating Centre for Health System and Financing); 2014.

[12] Diack A, Seiter A, Hawkins L, Dweik IS. Assessment of governance and corruption in the pharmaceutical sector: Lessons learned from low- and middle-income countries. 2010.

[13] World Health Organization. Global Health Expenditure Database. access on October 29, 2018.

[14] Menteri Kesehatan Republik Indonesia. Peraturan Menteri Kesehatan Republik Indonesia Nomor 48 Tahun 2013 tentang Petunjuk Pelaksanaan Pengadaan Obat dengan Prosedur E-Purchasing berdasarkan E-Catalogue.; 2013.

[15] Menteri Kesehatan Republik Indonesia. Peraturan Menteri Kesehatan Republik Indonesia Nomor 28 Tahun 2014 Tentang Pedoman Pelaksanaan Program Jaminan Kesehatan Nasional.; 2014.

[16] Menteri Kesehatan Republik Indonesia. Peraturan Menteri Kesehatan Republik Indonesia Nomor 63 Tahun 2014 tentang Pengadaan Obat Berdasarkan Katalog Elektronik (E-catalogue).; 2014.

[17] Kemetrian Kesehatan Republik Indonesia. Pedoman Penerapan Formularium Nasional. 2015.

[18] Dirjen Binfar dan Alkes Kemenkes RI. Keputusan Dirjen Binfar Nomor HK. 02. 03/ III/ 1346/ 2014 Tentang Pedoman Penerapan Formularium Nasiona. 2014.

[19] Menteri Kesehatan Republik Indonesia. Keputusan Menteri Kesehatan Republik Indonesia Nomor 171/ Menkes/ SK/ IV/ 2014 tentang Komite Penilaian Teknologi Kesehatan. 2014.

[20] Menteri Kesehatan Republik Indonesia. Keputusan Menteri Kesehatan RI No. HK. 02. 02/ Menkes/ 422/ 2016 tentang Komite Penilaian Teknologi Kesehatan. 2016.

[21] Presiden Republik Indonesia. Peraturan Presiden Republik Indonesia Nomor 12 Tahun 2013 tentang Jaminan Kesehatan.; 2013.

[22] Menteri Kesehatan Republik Indonesia. Pedoman Penerapan Formularium Nasional.; 2015.

[23] Niken Ariati. Tata Kelola Obat di Era Sistem Jaminan Kesehatan Nasional. 2017.

[24] Dirjen Binfar dan Alkes Kemenkes RI. Implementasi Formularium nasional dalam Pelaksanaan Jaminan Kesehatan nasional. 2015.

[25] Hidayat B, Mundiharno, Nemec J, Rabovskaja V, Rozanna C, Spatz J. Out-of-Pocket Payments in the National Health Insurance of Indonesia. 2015.

[26] Tong A, Sainsbury P, Craig J. Consolidated criteria for reporting qualitative research (COREQ): a 32-item checklist for interviews and focus groups. International journal for quality in health care 2007;19(6):349–357. 10.1093/intqhc/mzm042 17872937

[27] Mays N, Pope C. Qualitative research: Observational methods in health care settings. BMJ 1995 7 15;311(6998):182–184. 7613435 10.1136/bmj.311.6998.182 PMC2550229

[28] BPJS Kesehatan. Panduan Praktis Prolanis (Program Pengelolaan Penyakit Kronis). 2014.

[29] Hsieh H, Shannon SE. Three approaches to qualitative content analysis. Qual Health Res 2005;15(9):1277–1288. 10.1177/1049732305276687 16204405

[30] Menteri Kesehatan Republik Indonesia. Keputusan Menteri Kesehatan Nomor 1197/ Menkes/ SK/ X/ 2004, Standar Pelayanan Farmasi di Rumah Sakit. 2004.

[31] Menteri Kesehatan Republik Indonesia. Peraturan Menteri Kesehatan Nomor 58 Tahun 2014, Formularium Rumah Sakit. 2014.

[32] Kibuule D, Mubita M, Naikaku E, Kalemeera F, Godman BB, Sagwa E. An analysis of policies for cotrimoxazole, amoxicillin and azithromycin use in Namibia's public sector: Findings and therapeutic implications. Int J Clin Pract 2017;71(2): e12918. 10.1111/ijcp.12918 28090718

[33] Menteri Kesehatan Republik Indonesia. Keputusan Menteri Kesehatan Republik Indonesia Nomor 328/Menkes/IX/ 2013 tentang Formularium Nasional. 2013.

[34] Menteri Kesehatan Republik Indonesia. Keputusan Menteri Kesehatan Republik Indonesia Nomor HK.02.02/ Menkes/ 523/ 2015 tentang Formularium Nasional. 2015.

[35] Menteri Kesehatan Republik Indonesia. Keputusan Menteri Kesehatan Republik Indonesia Nomor HK. 01. 07/ Menkes/ 659/ 2017 tentang Formularium Nasional. 2017.

[36] Lembaga Kebijakan Pengadaan Barang dan Jasa Pemerintah (LKPP). Katalog Produk Obat 2018. Access on January 5, 2019;.

[37] Nguyen TA, Knight R, Roughead EE, Brooks G, Mant A. Policy options for pharmaceutical pricing and purchasing: issues for low-and middle-income countries. Health Policy Plan 2014;30(2):267–280. 10.1093/heapol/czt105 24425694

[38] Gustafsson LL, Wettermark B, Godman B, Andersén‐Karlsson E, Bergman U, Hasselström J, et al The ‘wise list’–a comprehensive concept to select, communicate and achieve adherence to recommendations of essential drugs in ambulatory care in Stockholm. Basic & clinical pharmacology & toxicology 2011;108(4):224–233. 21414143 10.1111/j.1742-7843.2011.00682.x

[39] Whyte P, Hall C. The Role of Health Technology Assessment in Medicine Pricing and Reimbursement Pharmaceutical Pricing Policies and Interventions.Geneva: WHO 2013.

[40] Hawkins L. WHO/HAI Project on Medicine Prices and Availability Review Series on Pharmaceutical Pricing Policies and Interventions. World Health Organization and Health Action International 2011.

[41] World Health Organization. WHO guideline on country pharmaceutical pricing policies.: World Health Organization; 2015.

[42] Jongudomsuk P, Samrit Srithamrongsawat, Walaiporn Patcharanarumol, Supon Limwattananon, Supasit Pannarunothai, Patama Vapatanavong, et al The Kingdom of Thailand Health System Review. 2015(Manila: WHO Regional Office for the Western Pacific).

[43] Babar Z. Pharmaceutical Policy in Countries with Developing Healthcare Systems.: Springer International Publishing AG; 2017.

[44] Jaafar S, Kamaliah Mohd Noh, Khairiyah Abdul Muttalib, Nour Hanah Othman, Judith Healy, Kalsom Maskon, et al. Malaysia health system review. 2013.

[45] Ministry of Health Malaysia. Malaysian National Medicines Policy 2nd Edition (Dasar Ubat Nasional (DUNas) Edisi Kedua 2012.

[46] Nguyen TA, Vitry A, Roughead EE. Pharmaceutical Policy in Vietnam Pharmaceutical Policy in Countries with Developing Healthcare Systems: Springer; 2017 p. 75–94.

[47] Huskamp HA, Deverka PA, Epstein AM, Epstein RS, McGuigan KA, Frank RG. The effect of incentive-based formularies on prescription-drug utilization and spending. N Engl J Med 2003;349(23):2224–2232. 10.1056/NEJMsa030954 14657430

[48] Menteri Kesehatan Republik Indonesia. Peraturan Menteri Kesehatan Republik Indonesia Nomor 30 Tahun 2014 tentang Standar Pelayanan Kefarmasian di Puskesmas. 2014.

[49] Doran T, Fullwood C, Gravelle H, Reeves D, Kontopantelis E, Hiroeh U, et al Pay-for-performance programs in family practices in the United Kingdom. N Engl J Med 2006;355(4):375–384. 10.1056/NEJMsa055505 16870916

[50] Leach M. Patients' views of pay for performance in primary care. Br J Gen Pract 2012 6;62(599):293. 10.3399/bjgp12X649061 PMC3361100 22687213

[51] Godman B, Wettermark B, van Woerkom M, Fraeyman J, Alvarez-Madrazo S, Berg C, et al Multiple policies to enhance prescribing efficiency for established medicines in Europe with a particular focus on demand-side measures: findings and future implications. Frontiers in pharmacology 2014; 5:106 10.3389/fphar.2014.00106 24987370 PMC4060455

[52] Wettermark B, Godman B, Jacobsson B, Haaijer-Ruskamp FM. Soft regulations in pharmaceutical policy making. Applied health economics and health policy 2009;7(3):137–147. 10.2165/11314810-000000000-00000 19799468

[53] Mashalla YJ, Sepako E, Setlhare V, Chuma M, Bulang M, Massele AY. Availability of guidelines and policy documents for enhancing performance of practitioners at the Primary Health Care (PHC) facilities in Gaborone, Tlokweng and Mogoditshane, Republic of Botswana. Journal of Public Health and Epidemiology 2016;8(8):127–135.

