# Supplementary material for: Use of medicine pricing and reimbursement policies for universal health coverage in Indonesia
Source: PLoS One. 2019 Feb 19;14(2):e0212328. doi: 10.1371/journal.pone.0212328 (PMC6380537; doi:10.1371/journal.pone.0212328)
Supplement: S1 Fig — (PDF) [file pone.0212328.s001.pdf]

S1 Fig. Conceptual framework

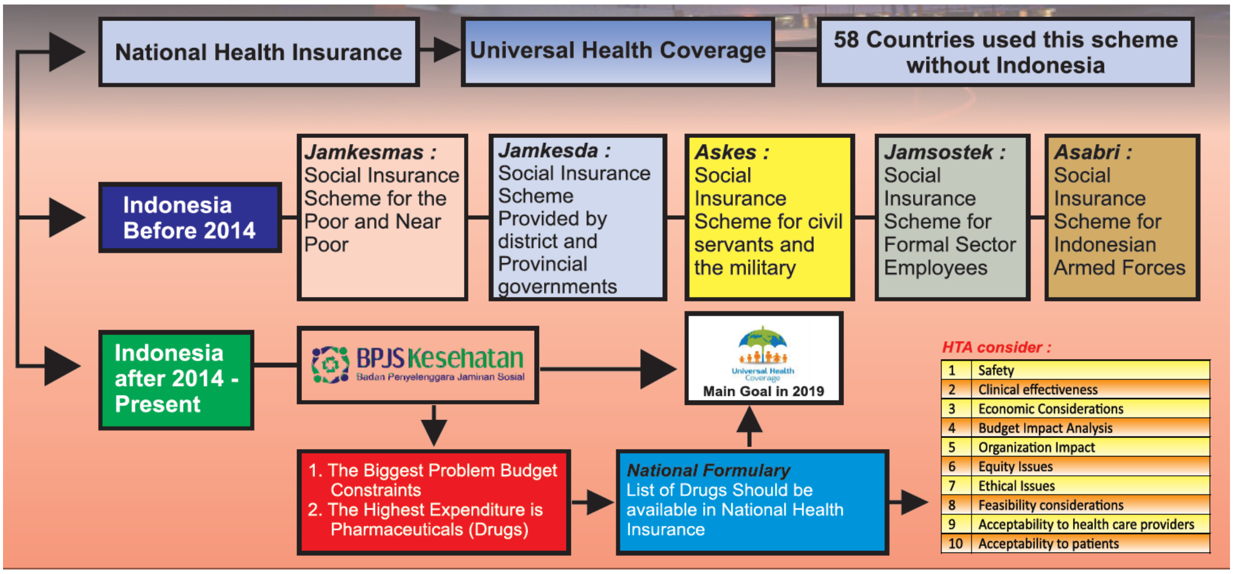

Explanation of conceptual framework:

Many countries aim to reform their national health insurance to reach universal health coverage. Nowadays, 58 countries are using universal health coverage besides Indonesia. Before 2014, various health insurance schemes covered health insurance for Indonesian society. As of 2014, all these schemes were integrated and organized under one agency called BPJS-Kesehatan. The main goal of this insurance is to achieve universal health coverage by 2019. The problem with BPJS-Kesehatan was budgetary constraints, with the highest expenditure being pharmaceuticals. The national formulary was used to organize medicines. HTA has not yet been applied in the national formulary, while many UHC countries do.
