# Supplementary material for: Use of medicine pricing and reimbursement policies for universal health coverage in Indonesia
Source: PLoS One. 2019 Feb 19;14(2):e0212328. doi: 10.1371/journal.pone.0212328 (PMC6380537; doi:10.1371/journal.pone.0212328)
Supplement: S1 Table — (PDF) [file pone.0212328.s002.pdf]

S1 Table. Initial list of themes and of questions

No	Themes	No	Questions
**1**	The development of medicine policy and national health insurance	1	Do you know how long the HTA Committee, NF Committee, and NHI Agency have existed?
		2	How many committee members?
		3	What are their professions?
		4	What are their duties?
		5	Why do they exist?
		6	What is their function?
		7	What is their role in supporting UHC?
		8	How is the NHI different from previous health insurance (Askes, Jamkesmas, etc)?
		9	How many people are covered by the NHI Agency currently?
**2**	Medicines policy for UHC	10	What medicines policy or policies should be created to support UHC?
		11	Are you involved in creating the NF and other medicines policies to support UHC?
**3**	Users Perspective	12	Do you know how the National Formulary is compiled?
		13	Do you agree with the NF?
		14	Do you have policy recommendations to maximize the use of HTA?
		15	Do you have other policy recommendations for prescribing drugs?
**4**	Obstruction and promoting factors of HTA	16	What is/are the advantage(s) using HTA in medicines policy?
		17	What is/are the disadvantage(s) using HTA in medicines policy?
		18	What is/are the facilitator(s) implementing HTA in medicines?
		19	What is/are the barrier(s) implementing HTA in medicines?
		20	What should other stakeholders do to solve them? And which ones?
		21	What should the government do?
**5**	The biggest burden of disease and medicine expenditure	22	What is the most widespread disease in Indonesia?
		23	How much money is allocated for medicines in the national health system?
		24	What medicines are allocated more money? (Why?)

**6**	Prescriptions not in NF	25	Have you ever received prescriptions for drugs not in the NF (How frequently and why)?
		26	Which medicines do you receive most often that are in the NF?
		27	Do you agree when doctors prescribe medicines not in the NF? Why?
