# Supplementary material for: Use of medicine pricing and reimbursement policies for universal health coverage in Indonesia
Source: PLoS One. 2019 Feb 19;14(2):e0212328. doi: 10.1371/journal.pone.0212328 (PMC6380537; doi:10.1371/journal.pone.0212328)
Supplement: S2 Table — (PDF) [file pone.0212328.s003.pdf]

S2 Table. Overview of recruitment process

TYPES OF STAKEHODERS	Send Letter	Respons	Interviews	Cancel after receiving list of questions	Stop The interview	No times available	Not response
WHO members	2	2	1	0		1	0
HTA Committee	3	2	2	0			1
NF Committee	3	1	1	0			2
BPJS Kesehatan (NHI Agency)	3	3	2	0		1	0
Pharmaceutical Industry	4	3	1	0		2	1
Physicians	12	10	6	1	3		2
Pharmacist	15	15	6	5	4		0
Patients	9	9	6	3			0
**TOTAL**	**51**	**45**	**25**	**9**	**7**	**4**	**6**
