# Supplementary material for: Use of medicine pricing and reimbursement policies for universal health coverage in Indonesia
Source: PLoS One. 2019 Feb 19;14(2):e0212328. doi: 10.1371/journal.pone.0212328 (PMC6380537; doi:10.1371/journal.pone.0212328)
Supplement: S3 Table — (PDF) [file pone.0212328.s004.pdf]

S3 Table. Final list of themes and aim of the themes

No	Themes	Aims
**1**	Knowledge of JKN-KIS program	To know the extent of participant's knowledge about the JKN-KIS program. Knowledge measured in this theme is the participant's knowledge about the agency, time execution, and the goals.
**2**	Attitude towards JKN-KIS program	To obtain participant perception and attitude of JKN-KIS program. Attitude measure in this theme from other personal information of participants.
**3**	Knowledge of medicines policy before JKN-KIS Program	To know the extent of participant's knowledge on medicines policy before JKN-KIS program. Such as List of Drug Price Platform (DPHO) in Askes, List of National Essential medicines (DOEN), etc.
**4**	Knowledge of medicines policy in the JKN-KIS program	To learn the participants' knowledge about the existence of e-Catalogue and the NF and their role.
**5**	Knowledge of the development of e-Catalogue and the NF	To find out the extent of the stakeholders' understanding of the process of developing the e-Catalogue and the NF.
**6**	Attitude towards e-Catalogue	To learn the participants' attitude about the existence of the NF
**7**	Attitude towards NF	To learn the participants' attitude about the existence of the NF
**8**	The Challenge of e-Catalogue and the NF	To learn about the challenges of both the e-Catalogue and the NF.
**9**	The Challenges of e-Catalogue	To learn about the challenges of the e-Catalogue.
**10**	The Challenges of the NF	To learn about the challenges of the NF.
**11**	Recommendation to improve e-Catalogue and the NF	To find out what the possible solutions are to address the challenges of the e-Catalogue and the NF.
**12**	Experience of prescribing not listed in the NF	To learn how frequently stakeholders, prescribe or received prescriptions not in medicine policies in the JKN-KIS program
**13**	Attitude towards prescribing not listed in the NF	To learn the participants' attitude concerning and towards prescriptions not in

		the NF
**14**	Reason for prescribing not listed in the NF	To know why prescriptions not in the NF occur.
**15**	Medicines not listed at the NF that mostly prescribed	To learn what non-NF medicines are prescribed the most.
**16**	Medicines listed at the NF that mostly unavailable	To learn what medicines are mostly unavailable in healthcare facilities.
**17**	Recommendation to avoid prescribing not listed in the NF	To learn what the stakeholder needs to avoid prescriptions not in medicine policies.
