# Supplementary material for: Use of medicine pricing and reimbursement policies for universal health coverage in Indonesia
Source: PLoS One. 2019 Feb 19;14(2):e0212328. doi: 10.1371/journal.pone.0212328 (PMC6380537; doi:10.1371/journal.pone.0212328)
Supplement: S1 File — (PDF) [file pone.0212328.s005.pdf]

S1 File. Final list of questions

List of questions for participants (excluding patients)

Key terms:

GOI	= Government of Indonesia
HTA	= Health Technology Assessment
JKN-KIS	= a National Health Insurance scheme of Indonesia
UHC	= Universal Health Coverage

1. What do you know about the JKN-KIS program? **Which points can you explain:**
   a. Implementing Agency;
   b. When executed;
   c. Implementation goals;
   d. Other personal information according to participants?
2. As far as you know, what public health insurance programs have been implemented prior to the implementation of the JKN-KIS program?
   a. Implementing Agency;
   b. The policy/program that they imposed to control medicines?
3. Do you know why all public health insurance programs are integrated into JKN-KIS?
4. What are the medicine policies/programs imposing on the JKN-KIS?
   a. The role or the functions of the policies
   b. What are the policy-making processes?
   c. Are you involved in the policy-making process?
   d. Who is involved in the policy-making process?
   e. Do you agree with the program/policies?
   f. Do you think there is/are stakeholder(s) harmed by that policy? For example, doctors, pharmacists, the pharmaceutical industry, patients, etc.
   g. Do you think the policy is based on the latest scientific evidence?
   h. In your opinion, are there any other policy proposals that should be applied?
5. What do you know about the HTA?
   a. Implementing Agency;
   b. When executed;
   c. Implementation goals;
6. Do you think the HTA should be applied to the medicine policies on the JKN-KIS?
7. In your opinion, what are the advantages and disadvantages of using HTA in medicine policies on JKN-KIS?
8. In your opinion, what are the supporting and inhibiting factors for the use of HTA on medicine policies in JKN-KIS?
9. In your opinion, what solutions should be found to implement the HTA on medicine policies in JKN-KIS?
   a. What should be done by all stakeholders (policymakers, doctors, pharmacists, drug companies, patients, etc.)
   b. What should be done by the GOI?
10. Have you ever found prescriptions not in the medicine policies on JKN-KIS?
    a. Do you know why this occurred?
    b. Do you agree with prescriptions not being in the medicines policy?

c. What medicines are most commonly prescribed that are not part of the policies of JKN-KIS?
d. Do you have any suggestions to overcome this?

11. Do you know how much money is allocated for medicines to support the JKN-KIS program?
    a. Do you know which drugs are allocated the largest budget?
12. Do you know what is the most widespread disease in Indonesia?
    a. Do you know how much money is allocated to cope with this disease?
13. Do you have other comments regarding this research?

List of Questions for Participants (excluding patients) in Bahasa

Pengertian Istilah:

HTA = *Health Technology Assessment*/ Penilaian Teknologi Kesehatan;
JKN-KIS = Program Jaminan Kesehatan Nasional di Indonesia;
UHC = Universal Health Coverage/ Cakupan Perlindungan Semesta.

1. Apa yang anda ketahui tentang JKN-KIS. **Point yang dapat anda jelaskan yaitu**:
    a. Instansi apa yang melaksanakan?
    b. Kapan mulai dilaksanakan?
    c. Apa tujuan dilaksanakan?
    d. Informasi lain yang dianggap perlu disampaikan dari partisipan
2. Sejauh yang anda ketahui, program jaminan kesehatan publik apa saja yang dilaksanakan sebelum diberlakukannya JKN-KIS?
    a. Instansi apa yang melaksanakan?
    b. Apakah anda tahu kebijakan/ program apa yang mereka terapkan untuk mengontrol obat?
3. Apakah anda tahu, mengapa seluruh program jaminan kesehatan publik berintegrasi ke JKN-KIS?
4. Apa sajakah kebijakan/ program yang sudah diberlakukan oleh pemerintah pada program JKN-KIS?
    a. Apakah anda tahu fungsi kebijakan/ program tersebut?
    b. Apakah anda tahu bagaimana proses pembuatan kebijakan tersebut?
    c. Apakah anda terlibat dalam proses pembuatan kebijakan tersebut?
    d. Siapa-siapa saja yang terlibat dalam proses pembuatan kebijakan tersebut?
    e. Apakah anda setuju dengan kebijakan tersebut? Mengapa?
    f. Menurut anda, adakah stakeholders (Dokter, Apoteker, Perusahaan obat, pasien, dll) yang dirugikan dengan kebijakan tersebut?
    g. Menurut anda, apakah kebijakan tersebut telah melalui bukti ilmiah mutakhir?
    h. Menurut anda, adakah usulan kebijakan lain yang sebaiknya diterapkan?
5. Apa yang anda ketahui tentang HTA?
    a. Instansi apa yang melaksanakan?
    b. Kapan mulai dilaksanakan?
    c. Apa tujuan dilaksanakan?
6. Menurut anda, apakah HTA sebaiknya diterapkan pada kebijakan obat yang sudah diterapkan pemerintah untuk mendukung JKN-KIS?
7. Menurut anda, apa keuntungan dan kerugian menggunakan HTA pada kebijakan obat yang sudah diterapkan pemerintah?
8. Menurut anda, apa faktor pendukung dan faktor penghambat menggunakan HTA pada kebijakan obat yang sudah diterapkan pemerintah?
9. Menurut anda, soulusi apa yang sebaiknya dilakukan untuk melaksanakan HTA pada kebijakan obat yang sudah diterapkan pemerintah?
    a. Apa yang sebaiknya dilakukan oleh para stakeholders (pembuat kebijakan, dokter, apoteker, perusahaan obat, pasien, dll)?
    b. Apa yang sebaiknya dilakukan oleh pemerintah?
10. Pernahkah anda menemukan peresepan obat di luar kebijakan obat yang sudah diterapkan

pemerintah?

   a. Apakah anda setuju dengan peresepan obat diluar kebijakan obat yang sudah diterapkan pemerintah?
   b. Obat apa yang paling sering diresepkan diluar kebijakan obat yang sudah diterapkan pemerintah?
   c. Apakah anda tahu, bagaimana cara mengatasi hal tersebut?

11. Apakah anda tahu berapa banyak anggaran belanja obat yang dialokasikan untuk mendukung JKN-KIS?
    a. Apakah anda tahu obat apa yang mendapatkan anggaran terbesar?
12. Apakah anda tahu penyakit yang terbesar di Indonesia?
    a. Apakah anda tahu berapa banyak anggaran untuk obat yang dialokasikan untuk mengatasi penyakit tersebut?
13. Apakah ada komentar lainyang ingin anda sampaikan terkait penelitian ini?

A List of Questions for Patients

**Key terms:**	
GOI	= Government of Indonesia
HTA	= Health Technology Assessment
JKN-KIS	= a National Health Insurance scheme of Indonesia
UHC	= Universal Health Coverage
NF	= National Formulary

1. Do you know about JKN-KIS, which is part of BPJS-Kesehatan? Could you explain briefly it?
2. Are you a participant in JKN-KIS?
3. When were you be a participant in JKN-KIS?
4. What health insurance did you use before JKN-KIS?
5. For how long were you a participant in that insurance?
6. Are you currently taking any medication?
    a. When was the last time you took the drugs?
    b. What drugs did you take?
7. Did you also take that medicine when using your previous insurance?
8. Do you feel a difference when taking medication of JKN-KIS compared with medication of your previous health insurance?
    a. Which one is cheaper?
    b. Which are more efficacious?
    c. Which one do you like, and think is better?
9. Is there a difference, do you feel, in terms of service between JKN-KIS and your previous health insurance?
    a. Are there differences when seeing doctors?
    b. Is there a difference when conducting laboratory checks or other checks?
10. Do you know about UHC? Could you explain briefly what you know?
    a. Do you agree with the GOI's target of reaching UHC in 2019?
    b. Do you think it can be achieved?
    c. Are there any suggestions from you in order to achieve the UHC?
11. Do you know about NF? Could you explain briefly about that?
    a. Do you know how NF is created?
    b. Do you agree with NF?
    c. What medications do you often receive which are in NF?
    d. Have you ever received prescriptions not in the NF? How often? Why?
    e. Do you agree with prescriptions not in the NF? Why?
    f. Do you have a policy recommendation in prescriptions?
12. Do you know about HTA? Could you explain briefly what you know?
    a. Do you think the HTA can be used for medicines policy in Indonesia?
    b. In your opinion, what is the advantage of using HTA in determining the choice of medicines?
    c. In your opinion, what are the disadvantages of using HTA in determining the choice of medicines?
    d. In your opinion, what are the promoting factors in implementing an HTA for drug selection?

e. In your opinion, what are the obstacles in implementing an HTA for drug selection?
f. In your opinion, what should be done by stakeholders to resolve this?
g. In your opinion, what should the government do?

13. Do you have suggestions for improving medicines policy?
14. Do you have other comments related to this research?

A List of Questions for Patients in Bahasa

1. Apakah anda tahu tentang jaminan kesehatan nasional yang dikelola oleh BPJS-Kesehatan? Bisakah anda Jelaskan sedikit tentang hal itu?
2. Apakah anda anggota BPJS-Kesehatan?
3. Kapan anda mulai menjadi anggota BPJS-Kesehatan?
4. Asuransi apa yang anda gunakan sebelum menjadi anggota BPJS-Kesehatan ?
5. Berapa lama anda menjadi anggota asuransi tersebut?
6. Apakah anda mengkonsumsi obat saat ini?
   a. Kapan terakhir kali anda mengkonsumsi obat?
   b. Obat apa?
7. Apakah obat tersebut juga anda konsumsi dengan menggunakan asuransi yang sebelumnya?
8. Apakah ada perbedaan yang anda rasakan ketika menggunakan obat dari BPJS dengan obat dari asuransi anda sebelumnya?
   a. Yang mana yang lebih murah?
   b. Yang mana yang lebih berkhasiat?
   c. Yang mana yang anda suka dan anggap lebih baik?
9. Apakah ada perbedaan yang anda rasakan dari sisi pelayanan pada Asuransi BPJS dan Asuransi yang anda gunakan sebelumnya?
   a. Apakah ada perbedaan ketika bertemu dengan dokter?
   b. Apakah ada perbedaan ketika melakukan cek laboratorium atau pemeriksaan lainnya?
10. Apakah anda tahu tentang Univeral Health Coverage (UHC)/ Jaminan kesehatan secara menyeluruh? Bisakah anda Jelaskan sedikit tentang hal itu?
    a. Apakah anda sependapat dengan pemerintah Indonesia yang ingin mencapai UHC di tahun 2019?
    b. Apakah anda pikir itu bisa dicapai?
    c. Apakah ada saran dari anda untuk mencapai UHC tersebut?
11. Apakah anda tahu tentang Formularium Nasional (Fornas)?
    a. Apakah anda tahu bagaimana Fornas dibuat?
    b. Apakah anda setuju dengan Formularium Nasional?
    c. Obat apa yang sering anda terima yang ada di dalam Fornas?
    d. Pernakah anda menerima obat di luar Fornas? Seberapa sering? Kenapa?
    e. Apakah anda setuju ketika dokter menuliskan obat diluar formularium nasional? Kenapa?
    f. Apakah anda memiliki rekomendasi kebijakan dalam penulisan obat?
12. Apakah anda tahu tentang Health Technology Assessment (HTA) / Penilaian Teknologi Kesehatan? Bisakah anda Jelaskan sedikit tentang hal itu?
    a. Menurut anda, apakah HTA dapat digunakan untuk kebijakan obat di Indonesia?
    b. Menurut anda, apa keuntungan menggunakan HTA dalam menentukan pilihan obat?
    c. Menurut anda, apa kerugian menggunakan HTA dalam menentukan pemilihan obat?
    d. Menurut anda, apa faktor pendukung dalam menjalankan HTA pada pemilihan obat?
    e. Menurut anda, apa faktor penghambat dalam menjalankan HTA pada pemilihan obat?
    f. Menurut anda, apa yang harus dilakukan oleh pemangku kebijakan untuk menyelesaikan itu?
    g. Menurut anda, apa yang harus dilakukan oleh pemerintah?
13. Apakah anda memiliki saran untuk meningkatkan kebijakan obat?
14. Apakah anda memiliki saran untuk penelitian ini?
